# Giant primary retroperitoneal myxofibrosarcoma mimicking myxoid liposarcoma: a diagnostic and surgical challenge

**DOI:** 10.1093/jscr/rjag919

**Published:** 2026-10-14

**Authors:** Amina Houmada, Zakaria El Mouatassim, Yassine El Bouazizi, Mohammed Reda El Hassouni, Oumayma Lahnaoui, Mohammed Anass Majbar, Amine Souadka

**Affiliations:** Gastrointestinal Oncology Surgery Department, Mohammed V Souissi University, Allal El Fassi Avenue, Rabat 10500, Rabat-Salé-Zemmour-Zaër, Morocco; Gastrointestinal Oncology Surgery Department, Mohammed V Souissi University, Allal El Fassi Avenue, Rabat 10500, Rabat-Salé-Zemmour-Zaër, Morocco; Gastrointestinal Oncology Surgery Department, Mohammed V Souissi University, Allal El Fassi Avenue, Rabat 10500, Rabat-Salé-Zemmour-Zaër, Morocco; Gastrointestinal Oncology Surgery Department, Mohammed V Souissi University, Allal El Fassi Avenue, Rabat 10500, Rabat-Salé-Zemmour-Zaër, Morocco; Gastrointestinal Oncology Surgery Department, Mohammed V Souissi University, Allal El Fassi Avenue, Rabat 10500, Rabat-Salé-Zemmour-Zaër, Morocco; Gastrointestinal Oncology Surgery Department, Mohammed V Souissi University, Allal El Fassi Avenue, Rabat 10500, Rabat-Salé-Zemmour-Zaër, Morocco; Gastrointestinal Oncology Surgery Department, Mohammed V Souissi University, Allal El Fassi Avenue, Rabat 10500, Rabat-Salé-Zemmour-Zaër, Morocco

**Keywords:** myxofibrosarcoma, retroperitoneal sarcoma, liposarcoma, MDM2, fluorescence *in situ* hybridization, compartmental resection

## Abstract

Myxofibrosarcoma (MFS) is a fibroblastic sarcoma that typically arises in the extremities of elderly patients; primary retroperitoneal involvement is exceptionally rare and often mimics myxoid liposarcoma. We report a 53-year-old man with a three-month history of progressive abdominal distension caused by a giant retroperitoneal mass measuring 26 cm. Core needle biopsy suggested a low-grade myxoid sarcoma, with myxoid liposarcoma not excluded. Multidisciplinary discussion led to en bloc left compartmental resection, including left colectomy, nephrectomy, adrenalectomy, and splenopancreatectomy. Histopathology confirmed a FNCLCC grade II myxoid sarcoma; fluorescence *in situ* hybridization showed no MDM2 amplification, confirming MFS and excluding dedifferentiated liposarcoma. The patient remained disease-free at 22 months. This case underscores the diagnostic overlap between retroperitoneal MFS and liposarcoma, the pivotal role of molecular pathology, and the importance of complete compartmental resection for local control.

## Introduction

Soft tissue sarcomas (STS) are rare malignant mesenchymal neoplasms representing ~1% of adult cancers [1]. Retroperitoneal sarcomas account for only 10%–20% of all STS and are often diagnosed at an advanced stage because of their deep anatomical location and nonspecific symptoms [2]. Myxofibrosarcoma (MFS) is a fibroblastic sarcoma characterized by abundant myxoid stroma, curvilinear vessels, and infiltrative growth. It predominantly arises in the extremities of elderly patients, whereas primary retroperitoneal involvement is exceptionally rare [3]. Unlike several other sarcomas, MFS lacks a specific immunohistochemical or molecular signature, making diagnosis challenging [4]. In the retroperitoneum, the main differential diagnosis is myxoid or dedifferentiated liposarcoma, particularly in large tumors with abundant myxoid stroma. Well-differentiated and dedifferentiated liposarcomas are characterized by amplification of the MDM2 and CDK4 genes on chromosome 12q13–15, making molecular analysis a key diagnostic tool [5, 6]. Complete en bloc resection with negative margins remains the cornerstone of curative treatment for retroperitoneal sarcomas [7, 8]. We report a giant primary retroperitoneal MFS initially suspected to be a myxoid liposarcoma, with definitive diagnosis established after surgery by fluorescence *in situ* hybridization (FISH).

## Case presentation

A 53-year-old man presented with progressive abdominal distension evolving over three months. He had no significant past medical history and had received no previous treatment. Physical examination revealed a large abdominal mass extending from the lower rib margin to the pubic region, measuring ~21 × 23 cm, associated with collateral venous circulation (Fig. 1). Laboratory tests were unremarkable.

**Figure 1:**
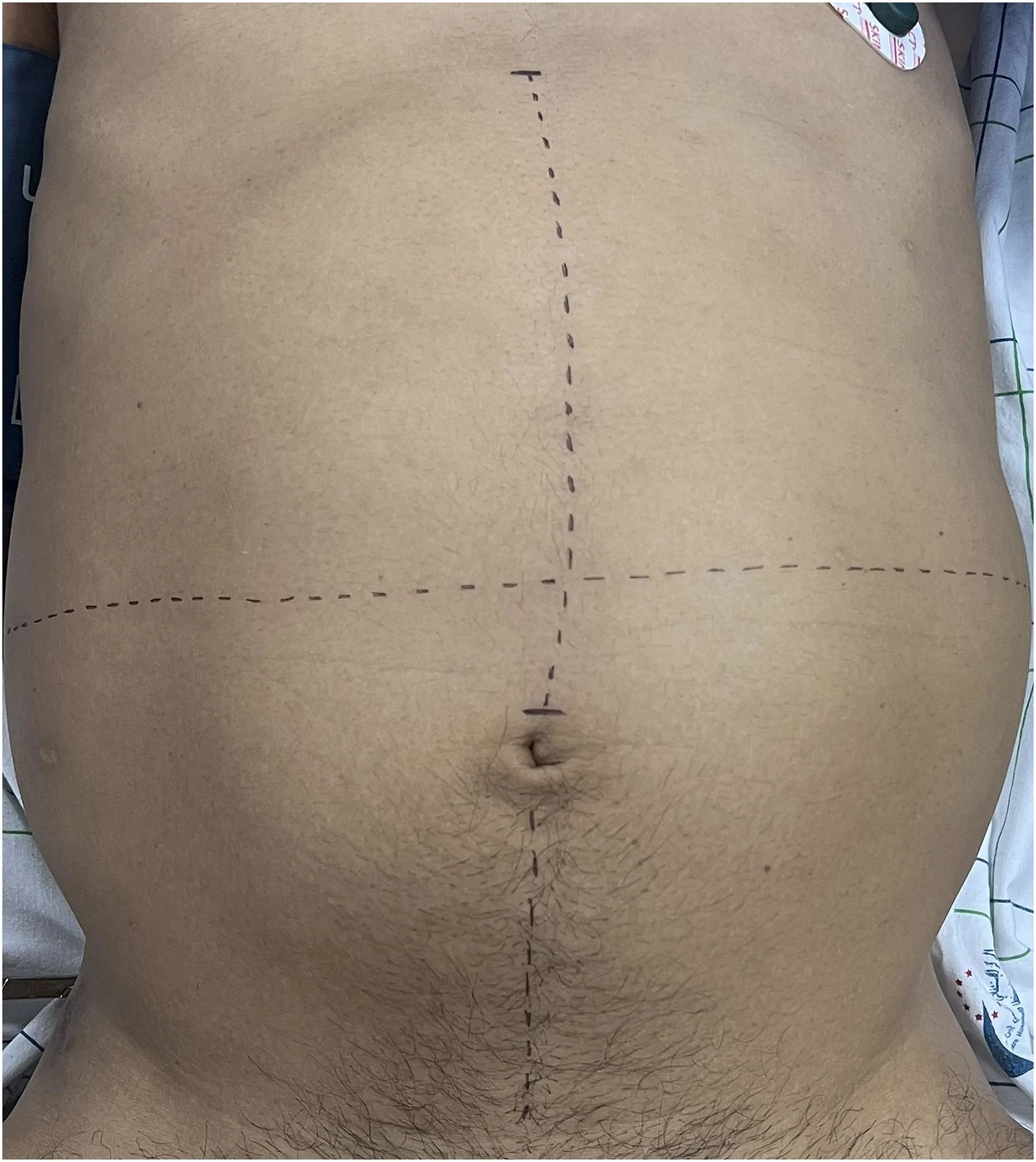
Preoperative clinical photograph showing marked abdominal distension caused by the giant retroperitoneal mass.

Abdominopelvic computed tomography (CT) angiography demonstrated a heterogeneous 26-cm intra-abdominal mass causing marked displacement of the stomach, pancreas, liver, and inferior vena cava. The tumor was closely related to the celiac axis, common hepatic artery, superior mesenteric vessels, and splenoportal confluence, without definite radiological evidence of vascular invasion (Fig. 2). Magnetic resonance imaging confirmed preservation of the fat planes and no clear adjacent organ invasion.

**Figure 2:**
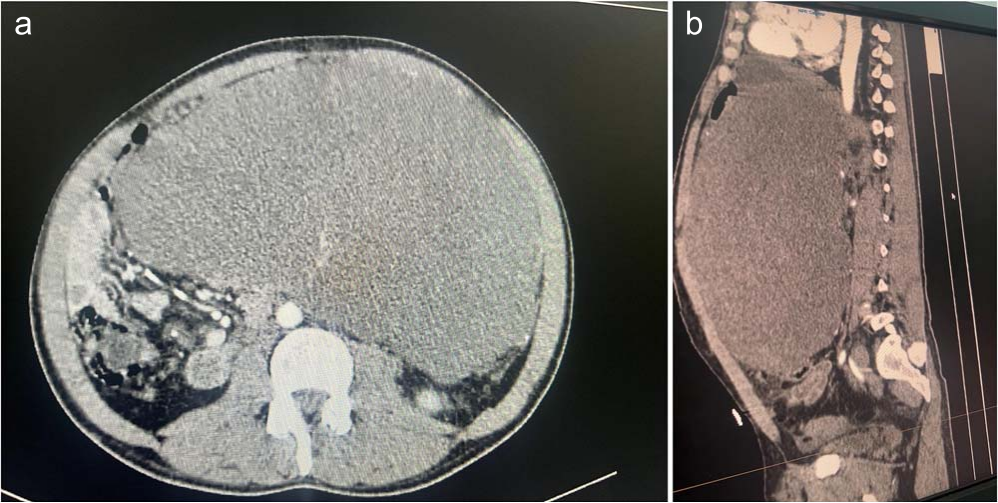
(a) Axial contrast-enhanced CT scan demonstrating the giant retroperitoneal mass. (b) Sagittal CT reconstruction illustrating the cranio-caudal extension of the lesion.

CT-guided core needle biopsy revealed a low-grade myxoid sarcomatous neoplasm, favoring MFS, although myxoid liposarcoma could not be excluded because of the retroperitoneal location. The case was discussed at a multidisciplinary sarcoma board, and surgical resection was recommended.

The procedure was performed through a xiphopubic midline laparotomy with bilateral transverse extensions. Intraoperative exploration revealed a massive tumor extending across the midline to the right and involving the spleen, distal pancreas, left colon, left kidney, and left adrenal gland, with close proximity to the celiac axis and the superior mesenteric artery.

The patient underwent left compartmental resection consisting of en bloc left colectomy, left nephrectomy with ipsilateral adrenalectomy, left splenopancreatectomy, and colorectal anastomosis (Figs 3 and 4).

**Figure 3:**
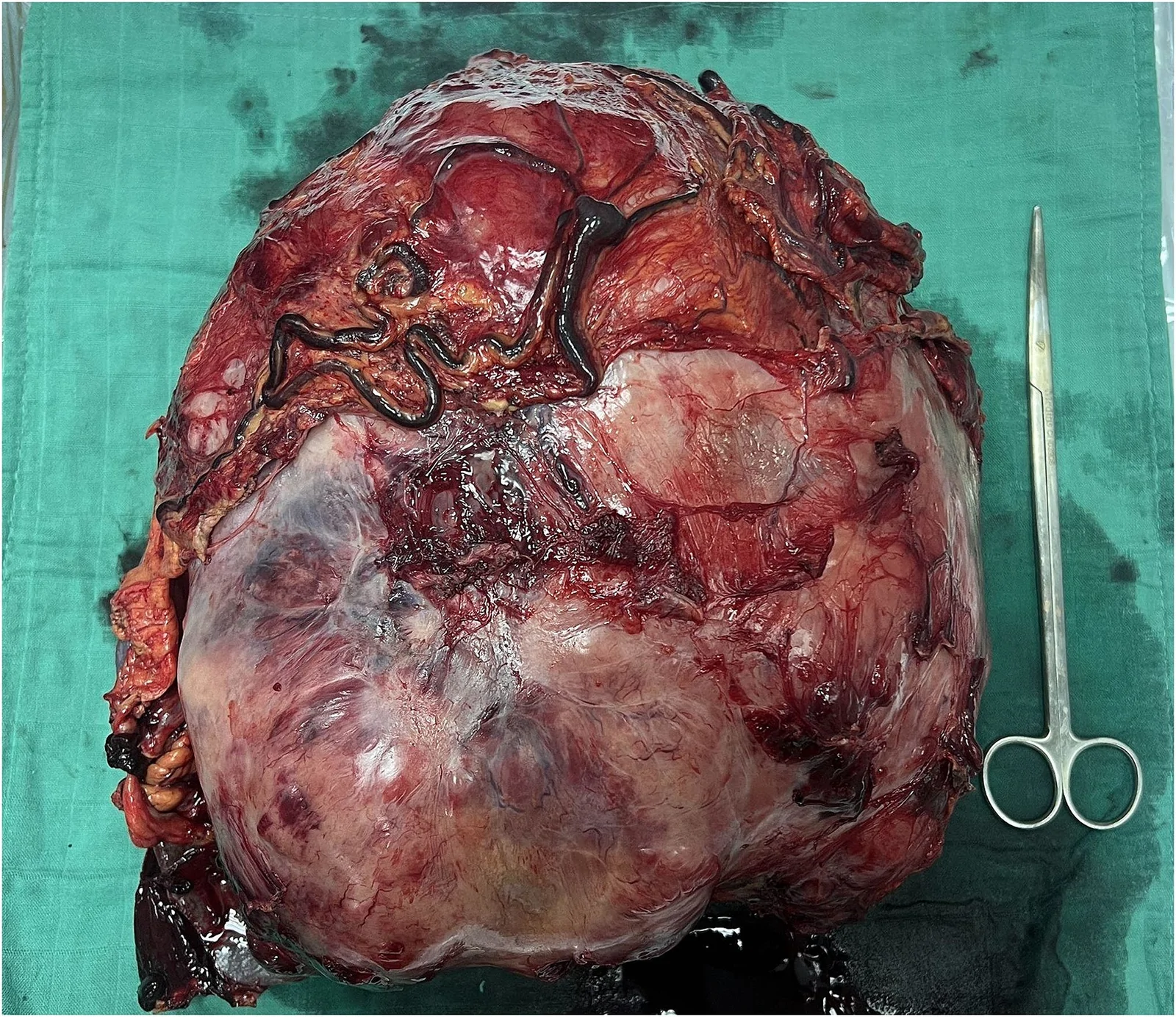
En bloc surgical specimen obtained after left compartmental resection, including the giant retroperitoneal tumor with multivisceral resection.

**Figure 4:**
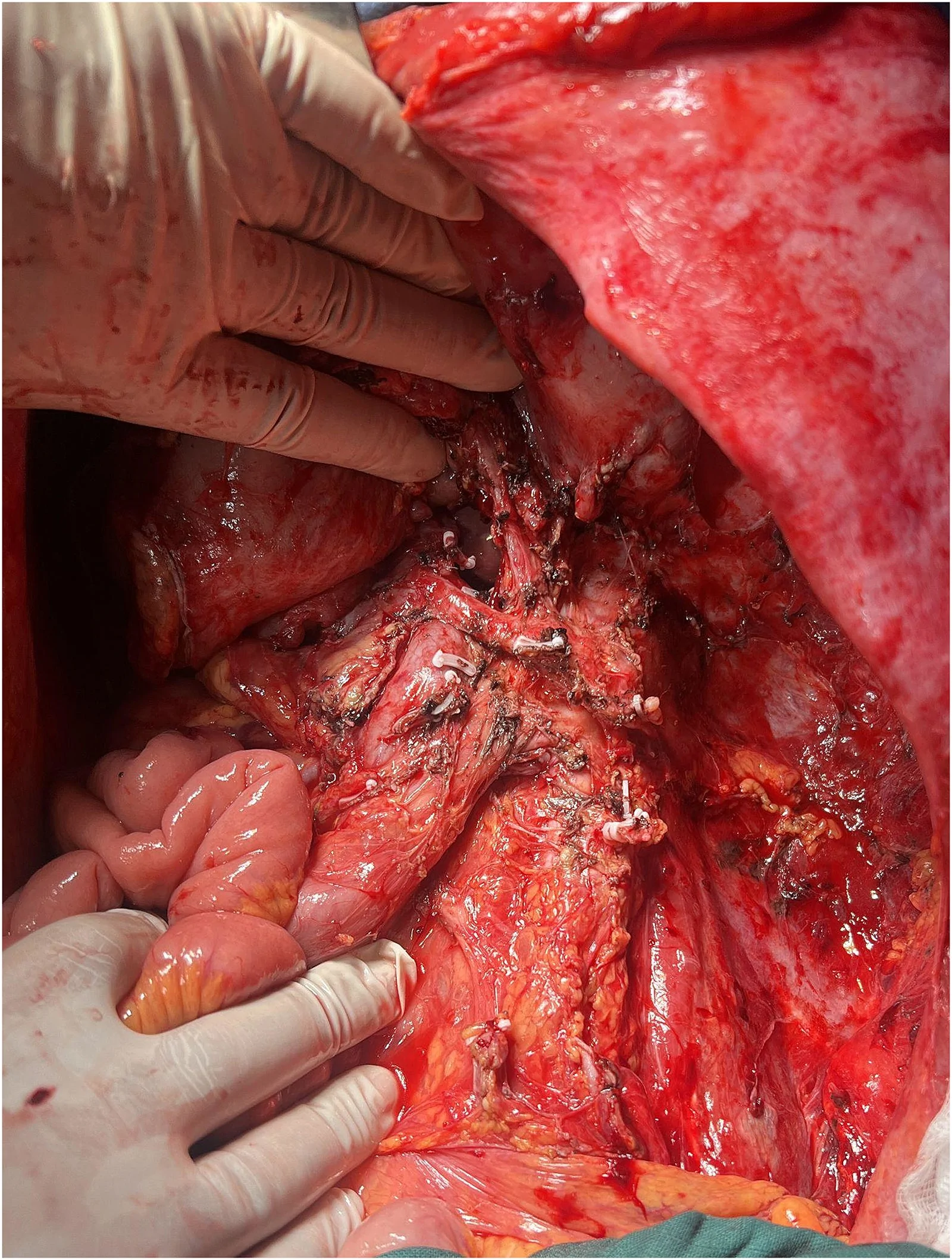
Intraoperative photograph after tumor resection demonstrating preservation of the superior mesenteric artery and vein following meticulous dissection.

The operation lasted ~5 hours, with an estimated blood loss of 300 ml. No intraoperative complications occurred, although meticulous dissection was required because of the tumor’s close adherence to the superior mesenteric artery and vein.

The postoperative course was complicated by a biochemical pancreatic fistula (ISGPS grade A), which resolved spontaneously with conservative management. The patient was discharged on postoperative day 14.

Histopathological examination showed a myxoid sarcomatous tumor, FNCLCC grade II (3 + 1 + 1), infiltrating the colonic serosa and subserosa, consistent with MFS. FISH analysis demonstrated absence of MDM2 amplification, confirming the diagnosis of MFS and excluding dedifferentiated liposarcoma. At 22 months of follow-up, the patient remained free of local recurrence or distant metastasis under regular clinical and radiological surveillance.

## Discussion

Primary retroperitoneal MFS is exceptionally uncommon, with only isolated cases described in the literature. Because of the large retroperitoneal space, these tumors may remain asymptomatic until they reach considerable dimensions, as observed in our patient [9, 10].

The major challenge in this case was the preoperative distinction between MFS and liposarcoma. Imaging findings were nonspecific, and core needle biopsy suggested a low-grade myxoid sarcoma without definitive classification. This reflects the well-recognized overlap between MFS and myxoid or dedifferentiated liposarcoma, which may share similar radiological and histological features. Furthermore, true primary retroperitoneal myxoid liposarcoma is extremely rare, and some authors consider many reported cases to represent dedifferentiated liposarcoma with prominent myxoid change [11].

Molecular analysis played a decisive role in our patient. Amplification of MDM2 and CDK4 is considered the molecular hallmark of well-differentiated and dedifferentiated liposarcoma [12]. The absence of MDM2 amplification on FISH strongly supported the diagnosis of MFS and effectively excluded dedifferentiated liposarcoma. This case therefore illustrates the complementary value of molecular pathology in the evaluation of diagnostically challenging retroperitoneal myxoid sarcomas [13].

Complete surgical resection remains the cornerstone of treatment for retroperitoneal sarcomas. Current evidence supports en bloc compartmental resection, including adjacent organs when necessary to achieve negative margins and improve local control. In our patient, multivisceral resection involving the spleen, distal pancreas, left colon, left kidney, and adrenal gland was required because of extensive compartmental involvement. Despite the close relationship with the superior mesenteric vessels and the celiac axis, complete macroscopic resection was achieved without vascular resection.

The postoperative biochemical pancreatic fistula resolved with conservative management, consistent with the generally favorable course of ISGPS grade A fistulas following distal pancreatectomy.

MFS is characterized by infiltrative growth and a high propensity for local recurrence, making adequate surgical margins and prolonged surveillance essential. The absence of recurrence at 22 months in our patient is encouraging, although continued long-term follow-up remains mandatory.

## Conclusion

This case highlights the diagnostic and therapeutic challenges of giant retroperitoneal myxoid sarcomas. The distinction between MFS and myxoid or dedifferentiated liposarcoma may remain uncertain after imaging and biopsy alone. In our patient, FISH analysis demonstrating absence of MDM2 amplification was crucial for confirming MFS and excluding dedifferentiated liposarcoma. Complete compartmental multivisceral resection, despite major vascular relationships, provided effective local control, and the patient remains disease-free 22 months after surgery.
